# High-Quality Draft Genome Sequence of an Endophytic *Pseudomonas viridiflava* Strain with Herbicidal Properties against Its Host, the Weed *Lepidium draba* L.

**DOI:** 10.1128/genomeA.01170-16

**Published:** 2016-10-20

**Authors:** Abdul Samad, Friederike Trognitz, Livio Antonielli, Stéphane Compant, Angela Sessitsch

**Affiliations:** AIT Austrian Institute of Technology GmbH, Bioresources Unit, Tulln, Austria

## Abstract

Here, we report the draft genome sequence of *Pseudomonas viridiflava* strain CDRTc14 a pectinolytic bacterium showing herbicidal activity, isolated from the root of *Lepidium draba* L. growing as a weed in an Austrian vineyard. The availability of this genome sequence allows us to investigate the genetic basis of plant–microbe interactions.

## GENOME ANNOUNCEMENT

The pectinolytic species *Pseudomonas viridiflava* represents one of the many phylogroups in the *P. syringae* species complex (1). It has a worldwide distribution and an extensive host range that includes numerous cultivated crops and weeds, including the model plant *Arabidopsis thaliana* (2, 3). This organism causes both compatible (disease) and incompatible (resistance) responses in *A. thaliana* that have triggered great interest in plant–pathogen interaction studies (3, 4).

*P. viridiflava* shows a high level of adaptability, both as a saprophyte and as a pathogen. Pathogenicity of this bacterium is correlated with the presence/absence of the *avrE* effector gene in two paralogous pathogenicity islands (T-PAI and S-PAI) of type III secretion systems (5). This bacterium was shown to display variability in phenotypic traits and genomic content, with all isolated *P. viridiflava* strains parting into two distinct phylogroups, causing disease symptoms of differing severities (6).

The *P. viridiflava* strain CDRTc14 was isolated from surface-sterilized roots of the weed *Lepidium draba*, sampled from an Austrian vineyard. This bacterium significantly inhibited germination and root growth of *L. draba in vitro* and under greenhouse conditions. It also shows indole acetic acid production and phosphate solubilization. Its genomic DNA was extracted following the standard phenol-chloroform method (7). Whole-genome shotgun sequencing was carried out on an Illumina HiSeq (GATC Biotech, Konstanz, Germany), producing 6,728,600 paired-end (2 × 125-bp) reads. Assembly was carried out with SPAdes version 3.8.0 (8). This resulted in 38 contigs (>500 bp), with a 221-fold mean coverage of a 5,963,467-bp genome length and yielded a contig *N*_50_ of 29,5098. One contig (67,392 bp) belonging to a plasmid was found by means of Blobtools (http://drl.github.io/blobtools/). The assembly quality was estimated in QUAST version 3.1 (9) and quality control of the mapping data was performed in Qualimap version 2.2 (10). PhyloSift version 1.0.1 (11) was used to verify the genome completeness, assessing a list of 40 highly conserved single-copy marker genes, all of which were found to be present.

Genome assembly was annotated using the NCBI Prokaryotic Genome Annotation Pipeline (12). The chromosome and plasmid of *P. viridiflava* CDRTc14 had GC contents of 59.31% and 55%, respectively. A total of 5,330 genes and 5,219 protein-coding genes were observed, along with seven genes encoding 5S rRNA, one gene encoding 16S rRNA, one gene encoding 23S rRNA, and 58 genes encoding tRNA.

Regarding pathogenicity, no complete pathogenicity islands or pathogenicity-related effector gene (*avrE*) was found in this strain. These results are consistent with a previous study showing *P. viridiflava* strain UASWS0038 as a valuable biological control agent due to the absence of pathogenicity islands (13). Strain CDRTc14 is equipped with several genes related to metal and semimetal resistance (54 genes), stress response (193 genes), and auxin biosynthesis (5 genes).

In-depth genomic comparison between *P. viridiflava* strain CDRTc14 and pathogenic strains will enable a better understanding of pathogen evolution and determinants of pathogenicity.

### Accession number(s).

The draft genome sequence for *P. viridiflava* strain CDRTc14 has been deposited in GenBank under the accession number MBPF00000000.
